# Correction

**DOI:** 10.1093/psyrad/kkac003

**Published:** 2022-03-10

In the originally published version of the below listed manuscripts, acknowledgement of NIH/NIMH funding was omitted in error. These errors have been corrected.

https://doi.org/10.1093/psyrad/kkab009

https://doi.org/10.1093/psyrad/kkab017

